# A Novel Variant in the *BICRA* Gene, Expanding the Phenotype: A Case Report

**DOI:** 10.1155/crig/4041217

**Published:** 2025-10-15

**Authors:** Catherine Kentros, Wendy K. Chung, Mythily Ganapathi

**Affiliations:** ^1^Department of Medical Genetics, Columbia University Medical Center, New York, New York, USA; ^2^Department of Pediatrics, Boston Children's Hospital, Boston, Massachusetts, USA; ^3^Department of Pathology and Cell Biology, Columbia University Medical Center, New York, New York, USA

**Keywords:** behavioral disorder, BICRA, bipolar type, Coffin–Siris syndrome 12, developmental delay, intellectual disability, microcephaly, psychiatric disorder, schizoaffective disorder

## Abstract

We report a 71-year-old female patient with a history of intellectual disabilities, dysmorphic features, schizoaffective disorder, bipolar type, and psychosis. Exome sequencing revealed a novel, heterozygous, predicted loss-of-function variant in the *BICRA* gene (NM_015711.3: c.1910del, p.[Leu637ArgfsTer87]). Heterozygous rare variants in *BICRA* have been associated with Coffin–Siris Syndrome 12 (CSS-12; OMIM #619325). This is the oldest patient to date with a pathogenic variant in *BICRA*. This case report expands the clinical phenotype associated with *BICRA* variants and sheds light on the long-term prognosis.

**Summary**

• Here, we report a novel variant in the *BICRA* gene associated with CSS-12.
• Although previous reports of patients with *BICRA*-related disorders highlight the intellectual disability, developmental delay, and behavioral issues in patients, this report expands the adult psychiatric phenotype to include schizoaffective disorder and bipolar type with accompanying psychosis.
• To our knowledge, a causal association between *BICRA* disease-causing variants and psychosis or psychotic features has not been established.
• Patients with reported disease-causing *BICRA* variants are primarily pediatric, and the onset of psychotic is usually in adults.
• Because all patients with *BICRA*-related disorders are younger than this patient, this case provides useful insight into the adult psychiatric phenotype.

## 1. Introduction

With the increased accessibility of comprehensive genomic testing, rare genetic etiologies of neurodevelopmental disorders and intellectual disability (ID) are being identified. The prevalence of ID in the general population is approximately 1%–3% [1]. In 2016, the worldwide prevalence of ID was 12.5 million [2]. So far, over 2500 genes associated with ID have been identified [3]. ID can be non-syndromic (NSID), presenting as an isolated feature, or syndromic (SID), presenting with dysmorphic features, structural anomalies, and/or multisystemic features. Psychiatric disorders are common comorbid conditions in individuals with ID. Some of the most prevalent chronic health conditions in children with ID are epilepsy, cerebral palsy, and anxiety disorder [4, 5]. With the exception of a few known recurrent conditions such as 22q11.2 deletion syndrome, the associations between monogenic conditions associated with developmental delay/ID in childhood and adult-onset psychiatric disorders such as psychosis and schizophrenia are not well understood. This is in part due to low utilization of genetic testing in certain practices such as adult psychiatric programs, and lack of long-term follow-up in adults.

Pathogenic and likely pathogenic variants in *BICRA* have been identified in patients with Coffin–Siris 12 [6], a cause of SID ID. Here, we report a patient with ID, adult-onset schizoaffective disorder and psychosis, with a likely pathogenic *BICRA* variant.

## 2. Materials and Methods

Informed written consent for personal data processing was obtained from patient's legal guardian. This study was approved by the Institutional Review Board of Columbia University Medical Center.

Duo exome sequencing was performed on the proband at Prevention Genetics with variant filtering and analyses as previously described [7].

## 3. Case Presentation

The proband is a 71-year-old female with a longstanding history of moderate to severe ID. The prenatal and early history were limited. Microcephaly was noted shortly after birth, as well as abnormal neonatal cry and colic. She developed a limited vocabulary of about 60 words. At 21 months of age, the patient underwent the Kuhlmann-Binet Intelligence test due to developmental delays. At the time, she was unable to sit without support, drink from a glass, or speak. She was found to have the mental age of an 11-month-old and an IQ score of 52, corresponding to moderate ID. The patient had several subsequent neuropsychological and IQ evaluations and scored consistently low, with limited speech and echolalia. At 18 years and 9 months of age, the Stanford-Binet intelligence test was repeated, revealing an IQ of 32. Cognitive testing was repeated at age 26, and results were similar to the previous scores in the range of severe ID. The patient was, at the time, on medication (unspecified) due to “head banging behaviors” and “tendency to act out.” She had occasional temper tantrums, staring off, completely dissociating, and fixating on her hands or her hair. Years later, these behaviors would be attributed to the patient responding to internal stimuli. No EEG, brain MRI, or metabolic results were available if they were performed.

By age 29, the patient was receiving 50 mg of thioridazine BID. She was in a sheltered workshop placement, in a loud setting. She started displaying episodes of screaming and staring at her hand and would appear to be talking to someone who was not there. These episodes would escalate to self-injurious behaviors. At the age of 35, thioridazine was discontinued following a gradual wean. Within 3 weeks of discontinuation, the emergence of outbursts and agitation was reported. A behavior support plan was developed to address this behavioral change. These outbursts occurred one to three times per month, lasting between 2 and 45 minutes. Interventions included attempts to restrain her and take her to an isolated room. Thioridazine was restarted, which resulted in the de-escalation of behavioral issues. At 35 years and 11 months, re-emergence of disruptive behaviors occurred, including disrobing and urinating on floors. The patient was evaluated by a psychiatrist, who recommended a neurological workup to rule out temporal lobe seizures, but follow-up documentation is unavailable. The patient was started on haloperidol, and thioridazine was discontinued. In the next 2 months, reports of excessive self-stimulatory behaviors were noted. The patient was thought to be allergic to haloperidol and was switched back to thioridazine. At age 38, the patient had a psychological reassessment, and her clinical picture became more indicative of psychosis; she began to refer to herself in the third person and would give commands to herself in the third person. She would often talk or yell at her hand. The patient would display mood swings that would manifest as irritability and emotional lability, as well as auditory and visual hallucinations. Negative symptoms of schizophrenia were also noted.

By age 53, the patient was started on olanzapine at bedtime, and thioridazine was discontinued. Olanzapine was noted to be highly effective in managing her behaviors, including a marked reduction in response to internal stimuli and a decrease in agitation. At 54 years, olanzapine was discontinued for unknown reasons, and agitation re-emerged. The patient was started on oxcarbazepine. The patient began talking to herself incessantly. Staff at her program noted an increase in aggression. The patient started clenching her hands into fists and demonstrated rapid eye-blinking. Divalproex sodium 500 mg BID was initiated. Oxcarbazepine was discontinued as it was deemed ineffective. A few months later, divalproex sodium was discontinued due to the emergence of manic behaviors. Olanzapine was reintroduced and aggressive episodes were significantly reduced. At age 58, the patient was taking olanzapine 10 mg QD and lorazepam 0.5 mg PRN to help with insomnia. By this time, self-injurious behaviors had subsided. Episodes of talking to herself, her hand, the ceiling, or the wall would happen occasionally, but not nearly as often as before starting olanzapine. Psychiatry notes started referencing the diagnoses of schizoaffective disorder, bipolar type, and psychosis. The patient displayed auditory hallucinations and mood swings. Prior to the start of olanzapine, the patient would also display negative symptoms of schizophrenia (monotonic speech, lack of eye contact, lack of gesturing, and social withdrawal), all of which were reduced while on olanzapine.

In her late 60s, episodes of talking to herself and responding to internal stimuli still occasionally occurred, though her psychiatric symptoms appeared to have been best managed on olanzapine. The patient died of a myocardial infarction at age 71. To our knowledge, there are no documented cardiovascular phenotypes associated with *BICRA* variants. As described in a meta-analysis conducted by Yu et al. [8], myocardial infarction may be linked to long-term antipsychotic use.

In addition to having many overlapping clinical features with the previously reported CSS-12 patients, this patient's case expands the adult psychiatric phenotype. The patient was notably dysmorphic. Her physical features included bilateral arm deformity, thin upper and lower lips, high hairline, epicanthal folds, bilaterally low-set ears, bulbous nasal tip, and prominent nasal bridge. Her history was notable for short stature (adult height of 4 feet 9 inches). The patient shared many of the features described in the literature (Table 1). The patient was able to state and write her full name, read basic words, identify certain colors, and match objects to pictures.

**Figure 1:**
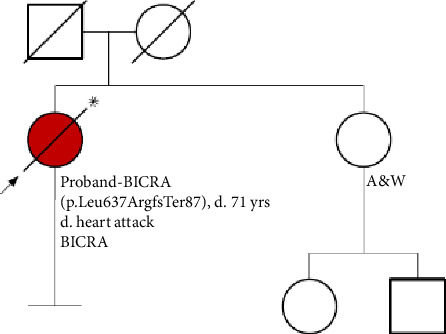
Proband's family pedigree.

The patient had one full biological sibling (Figure 1). Her sister and both parents were developmentally appropriate. There is no notable history of psychiatric disorder, autism, developmental delay, or ID in the immediate family. The family is of European ancestry.

## 4. Results

Singleton exome sequencing revealed a novel, likely pathogenic, rare, loss-of-function variant in the *BICRA* gene (NM_015711.3: c.1910del, p.[Leu637ArgfsTer87]) in the coding exon 4 of 13. Inheritance of the variant is unknown, as the parental samples are unavailable (parents are deceased). Cases with loss of function variants downstream of this variant have been reported [9, 10]. No other genetic testing was performed.

## 5. Discussion

At the time of this report, 15 individuals have been published with BICRA-related disorders [10–12]. Their shared phenotypes include developmental delay, ID, autism spectrum disorder, behavioral issues, microcephaly, and dysmorphic features. Out of the 15 individuals reported, 13 were heterozygous for predicted loss-of-function variants and 2 for missense variants.

As noted by Asadauskaitė et al. and Barish et al., emotional outbursts, irritability, impulsivity, echolalia, labile moods, and oppositionality have been noted in patients with *BICRA*-related disorders. The psychiatric history of these patients, however, has not been followed longitudinally. The clinical findings of this case confirm the findings previously associated with BICRA-related disorders, including the cognitive phenotype, craniofacial findings, and short stature, yet further expand the psychiatric phenotype through a long life course. Besides our patient, the oldest patient reported was 28 years old and no adult-onset psychiatric illness was reported [10].

## Figures and Tables

**Table 1:** Medical and developmental clinical findings in BICRA patients described in the literature.

	Our case	Tu et al. case report	Asadauskaitė et al. case report	Barish et al.											
			P1	S1	S2	S3	S4	S5	S6	S7	S8	S9	S10	S11	S12
cDNA Variant (NM_)	c.1910del	c.1666C > T	c.535C > T	c.936delC	c.1574delG	c.1993C > T	c.2075_2078delCTCA	c.2479_2480delinsA	c.3247dupT	c.4369C > T	∼126 kb loss	∼167.8 kb loss	∼202 kb loss	c.192G > C	c.4267G > A
Amino acid change	p.(Leu637Argfs∗87)	p.(Gln556∗)	p.(Gln179∗)	p.(Ala313Profs∗30)	p.(Ser525Thrfs∗199)	p.(Gln665∗)	p.(Thr692Argfs∗31)	p.(Ala827Thrfs∗15)	p.(Cys1083fs∗26)	p.(Gln1457∗)	Deletion	Deletion	Deletion	p.(Glu64Asp)	p.(Glu1423Lys)
Inheritance	Unknown, presumed de novo	De novo	De novo	De novo	Non-maternal	De novo	De novo	De novo	De novo	De novo	De novo	De novo	De novo	De novo	De novo
Age	70 y	25 mos	5 y	3 y	8 y	11 y	9 mos	17 y	28 y	17 y	2 y	7 y	8 y	11 y	28 mos
Sex	F	F	M	F	F	M	M	M	M	M	M	F	F	M	F
DD/ID	Y	Y	Y	Y	Y	Y	Y	Y	Y	Y	Y	Y	Y	Y	Y
Behavioral problems (Y/N)	Y	N	Y	N	N	Y	N	N	Y	Y	N	N	Y	Y	N
Psychiatric	Schizoaffective disorder, bipolar type psychosis	—	—	—	—	—	—	—	—	—	—	—	—	—	—
Growth (SS, FTT, PWG)	SS	FTT	FTT	PWG	SS		PWG	SS, PWG, FTT			PWG, FTT				SS, PWG, FTT
Dysmorphic features (Y/N)	Y	N	Y	Y	Y	Y	Y	Y	Y	Y	Y	Y	Y	Y	Y
Microcephalic (Y/N)	Y	N	Y	—	Y	—	—	Y	—	—	—	Y	Y	Y	Y
Hernia	Inguinal hernia	—	Inguinal hernia	—	—	—	—	—	—	—	—	—	—	—	—

*Note:* (Y/N) = yes/no.

Abbreviations: DD/ID = developmental delay, intellectual disability, FTT = failure to thrive, PWG = poor weight gain, SS = short stature.

## Data Availability

The data and clinical information used for the writing of this report is provided herein in this case report. The variant was deposited in ClinVar (accession SCV004809185).
